# Response to therapy in Richter syndrome: a systematic review with meta-analysis of early clinical trials

**DOI:** 10.3389/fimmu.2023.1295293

**Published:** 2023-11-23

**Authors:** Mário Sousa-Pimenta, Ângelo Martins, José Mário Mariz, Pedro Berraondo

**Affiliations:** ^1^ Department of Hematology and Bone Marrow Transplantation, Portuguese Oncology Institute of Porto (IPO-Porto), Porto, Portugal; ^2^ i3S - Instituto de Investigação e Inovação em Saúde, Universidade do Porto, Porto, Portugal; ^3^ Department of Biomedicine, Unit of Pharmacology and Therapeutics, Faculty of Medicine, University of Porto, Porto, Portugal; ^4^ Department of Immunology and Immunotherapy, Cima Universidad de Navarra, Pamplona, Spain; ^5^ Navarra Institute for Health Research (IDISNA), Pamplona, Spain; ^6^ Centro de Investigación Biomédica en Red de Cáncer (CIBERONC), Madrid, Spain

**Keywords:** Richter syndrome, immunotherapy, targeted-therapy, cell-based therapy, meta-analysis

## Abstract

**Introduction and aims:**

Richter syndrome (RS) represents the clonal evolution of chronic lymphocytic leukemia with histological transformation into a high-grade B cell lymphoma (diffuse large B cell lymphoma - DLBCL) or Hodgkin lymphoma. Considering that RS is an uncommon condition with poor prognosis, few high-quality evidence is available. To overcome this unmet need, this meta-analysis aimed to pool efficacy of early clinical trials in Richter syndrome (DLBCL subtype).

**Methods:**

MEDLINE, Scopus and Web of Science were searched up to May of 2023 to identify clinical trials decoying efficacy. The pooled complete response, objective response and intension-to-treat failure rates were calculated by pharmacological categories (classical chemotherapy, immunochemotherapy, immunotherapy, Bruton-tyrosine kinase inhibitors, targeted approaches, cell-based therapies and combinatorial regimens) using the Der-Simonian and Laird random-effects model. The Freeman-Tukey double arcsine method was used to estimate variance and confidence intervals. Heterogeneity was assessed using the I^2^ method.

**Results:**

Overall, from 1242 studies identified, 30 were included, pooling data from 509 patients. The higher efficacy rates when, cell-based therapies were excluded, were achieved by immunochemotherapeutic regimens followed by combinatorial regimens, with complete response rates of 21.54% (IC95%14.93-28.87) and 23.77% (IC95% 8.70-42.19), respectively. Bispecific antibodies (alone or coupled with a chemotherapy debulking strategy) overtook Bruton tyrosine kinase inhibitors response rates. The latter, although achieving objective response rates above average, presented scarce complete response rates. Checkpoint inhibitors alone usually do not lead to complete responses, but their effectiveness may improve when combined with other agents, unveiling the importance of immune microenvironmental modulation.

**Conclusion:**

This is the first meta-analysis of early clinical trials assessing the impact of different therapeutics in RS. By analyzing the pooled efficacy estimates, our work suggests the role of a tailor-made bridging therapy for young patients with RS eligible for allogeneic hematopoietic stem cell transplantation (alloSCT), formally the only curative strategy.

## 1 Introduction

Richter syndrome affects 2 to 10% of patients diagnosed with chronic lymphocytic leukemia (1), being regarded as the phenotypic transdifferentiating phenomenon that occurs in a minority of chronic lymphocytic leukemia patients who develop a diffuse large B-cell lymphoma (DLBCL) or, less commonly, Hodgkin’s lymphoma (2). If clonally related to chronic lymphocytic leukemia, RS has a worse prognosis in comparison with clonally unrelated clones (3, 4). Indeed, while its etiopathogenesis involves a gradual accumulation of genomic and microenvironmental changes resulting in clonal divergence, the debate surrounding the presence or absence of clonality in relation to CLL vividly underscores the uncertainties regarding the cell of origin in RS. It remains unclear whether RS originates from CLL cells with stem-like properties or from dormant circulating CLL pools that, through successive damage, achieve competitive edge and become predominant (5). The median overall survival of patients diagnosed with RS rounds 12 months; however, this expectancy decreases with the presence of other risk factors or previous exposure to treatment regimens (6). Several risk factors have been associated with RS burden, including unmutated immunoglobulin heavy-chain status, TP53 pathological variants, BCR stereotype subset #8, del(17p) or complex karyotype, NOTCH1 and MYC mutations, as well as CDKN2A/B tumour suppressor gene loss (5).

Concerning the immunological fitness unbalance in CLL, which preceeds overt RS burden, there is an inhibitory effect in NK cells cytotoxicity, decreased function of γδ T cells, increased T cell exhaustion and higher circulating T regulatory (Treg) lymphocytes (7). Impaired immune effector functions and increased immune tolerance may jeopardize the immunosurveillance, creating a substrate for disease progression. Beyond immunological overall fitness, nodal tissue analysis revealed a higher PD-L1 expression in histiocytes and dendritic cells of RS patients in comparison to those with CLL, analogously to increased infiltration by FOXP3^+^ T cells and CD163-positive macrophages (8). These results not only endorse the idea of maintaining an immune microenvironment that nurtures malignancy but also underscore the prerequisites for the implementation of immunotherapeutic approaches with checkpoint inhibitors.

The efficient treatment of RS comprises a deep understanding of the disease biology and patient characteristics (namely immunological fitness status, highly dependent upon previous therapeutic incursions for CLL and/or RS), as well as a strategical therapeutic sequencing that maximizes the potential to bridge young and fit patients to allogeneic hematopoietic stem cells transplantation. Beyond R-CHOP (rituximab plus cyclophosphamide, doxorubicin, vincristine, and prednisone) as a frontline approach to RS-DLBCL subtype and the use of platinum-based regimens as rescue therapy (1), there is no established consensus regarding the therapeutic sequencing.

This systematic review and meta-analysis delves into the treatment approaches for RS (DLBCL-subtype) that were the focus of clinical trials, aiming to assess their efficacy (complete response and objective response rates). By comparing the outcomes of early trials and analyzing the characteristics of enrolled patients, we intend to suggest a tailor-made therapeutic sequencing.

## 2 Methods

### 2.1 Literature search

This study was conducted according to the Cochrane collaboration guidelines for systematic reviews. The search was performed in MEDLINE (https://pubmed.ncbi.nlm.nih.gov), Web of Science (https://www.webofknowledge.com) and SCOPUS (https://www.scopus.com). All data, without language or publication dates restriction imposed, was collected from inception up to 7^th^ May of 2023 using the following keywords or medical subject heading terms: “Richter” AND (“syndrome” OR “transformation”) AND (“treatment” OR “therapy”).

### 2.2 Eligibility criteria

We aimed to identify all relevant publications focusing clinical trials assessing the efficacy of treatment strategies in the context of Richter syndrome. Only scientific publications that fulfill the inclusion criteria were analyzed, namely: 1) clinical trials addressing therapeutic approaches to Richter syndrome (classical chemotherapeutic agents, targeted drugs, immunomodulatory agents and/or cell-based therapies); 2) all patients should have active disease upon study enrollment and 3) studies had to report at least complete response and/or partial response rates; 4) studies should provide efficacy results of patient treated with doses below the toxicity threshold.

The exclusion criteria were: being case reports, observational studies, narrative reviews, *in vitro* assays and animal studies, as well as guidelines, editorial, correspondences, consensus statements and cost-effectiveness studies, which were withdrawn from analysis.

### 2.3 Data collection

Two authors (MSP and AM) independently reviewed the titles and abstracts of the studies identified in the search and excluded those that clearly did not meet the eligibility criteria. The full text of the remaining manuscripts was evaluated to determine their inclusion or exclusion. The lists of studies selected for inclusion independently by each author were compared, and disagreements were resolved jointly through discussion until consensus was reached; when necessary, a third author was also involved in the discussion. The following information was abstracted from each study into a data extraction form: complete response rate, objective response rate (the sum of complete and partial response rates), prevalence of the most frequent and severe (grade ≥ 3) adverse effects. Differences in data extraction were settled by consensus.

### 2.4 Quality assessment

The methodology of studies and the reporting quality were assessed independently by two authors using the Critical Appraisal Skills Programme (CASP) checklist for Clinical Trials, adapted considering the inclusion of early clinical trials that preclude randomization. Furthermore, an analysis of potential bias across the studies was performed by examining funnel plots to identify any signs of asymmetry.

### 2.5 Statistical analysis

The endpoints analyzed were: i) objective clinical response, ii) complete response rates, and iii) treatment failure in intension-to-treat analysis (patients not achieving at least a partial response, maintaining a stable disease or progressing) of the patients with Richter syndrome submitted to pharmacological therapies. The proportions achieved at each outcome were retrieved from each study and the pooled metrics were obtained using the Der-Simonian and Laird random-effects model, which considers both between-study and within-study variation (9, 10). The Freeman–Tukey double arcsine method was used to retrieve the variance, as previously reported (11). Furthermore, subgroup analysis was performed, dividing studies by the most common therapeutic strategies (classical chemotherapy regimens; chemoimmunotherapy [monoclonal antibodies and/or immunomodulators and/or bispecific antibodies coupled with classic chemotherapy backbones]; immunotherapy [bispecific antibodies or immune-checkpoint inhibitors]; Bruton-tyrosine kinase inhibitors [iBTK]; other targeted therapies excluding iBTK and cell-based therapies, comprising CAR-T cells). The evaluation of heterogeneity among the studies was conducted through I^2^ statistics along with corresponding 95% confidence intervals. Lastly, Egger’s statistical tests followed by the funnel plot visual assessment were used to recognize publication bias. All statistical analyses were performed using the meta package (12) for R, version 3.5.3 (R Foundation for Statistical Computing, Vienna, Austria). A p-value less than 0.05 was considered statistically significant.

## 3 Results

### 3.1 Selection of studies and analysis of the reporting quality

**Figure 1:**
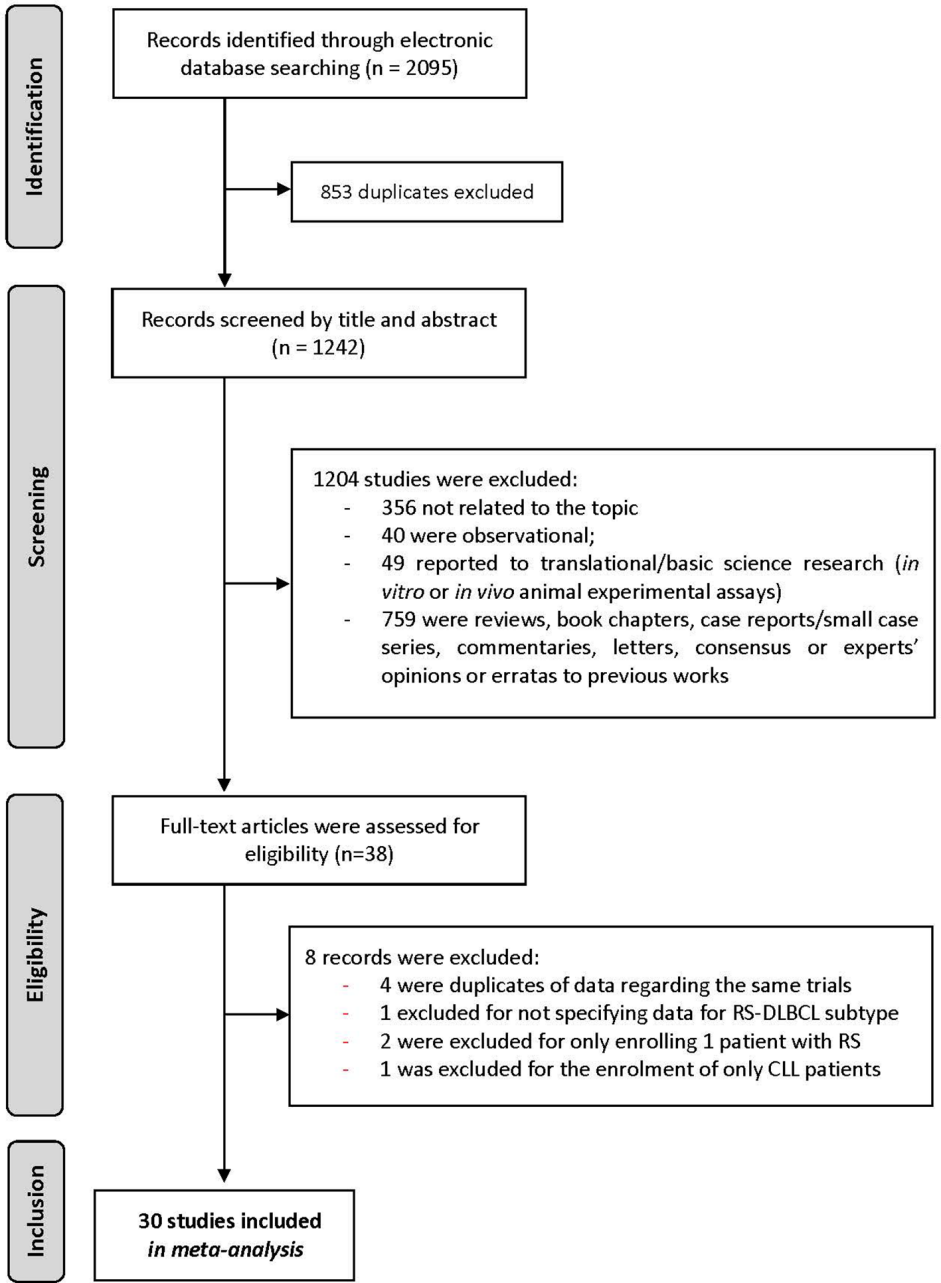
Flowchart representing the selection of studies.

Our query identified 2095 records, of which 853 were duplicates. The remaining 1242 articles were evaluated considering the titles and abstracts, and 1204 records that did not fulfill the inclusion criteria were eliminated. Then, 38 potentially relevant records were retrieved and underwent full evaluation. Finally, 30 studies were included in our meta-analysis (**Figure 1**).

**Figure 2:**
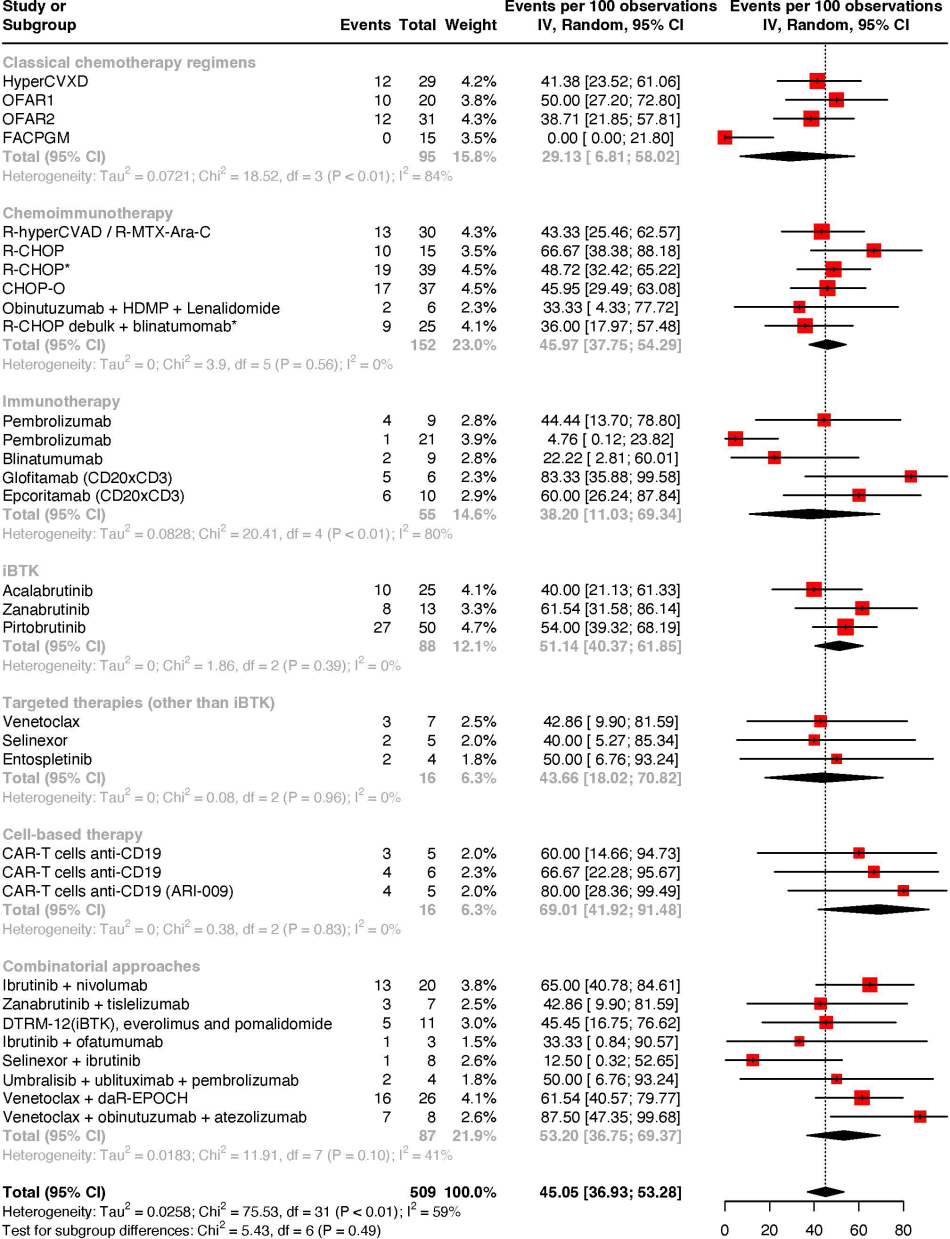
Meta-analysis of objective response rates by treatment strategies.

The analysis of studies was performed following the Comprehensive Assessment of Study Protocols (CASP) for clinical trials. Overall studies’ quality was classified as moderate (**Figure 1**, **Supplementary Materials**). The analysis of the funnel plots along with the results of the Egger test (p>0.05) suggested low potential for publication bias (**Figure 2**, **Supplementary Materials**).

### 3.2 Characteristics of the studies

Characteristics of studies are summarized in **Table 1**. Thirty studies were included, comprising early phase clinical trials (phase I and II) assessing response’ efficacy of therapeutic approaches in Richter syndrome, DLBCL-subtype. From these, four assessed the outcomes with classical chemotherapy regimens, five explored immunochemotherapy regimens, five focused on immunotherapy as a standalone approach, three dissected regimens encompassing Bruton-tyrosine kinase inhibitors and three studies analyzed other targeted therapies beyond iBTK, eight studies assessed combinatorial approaches and three analyzed the outcomes of CAR-T cells. Globally, the studies enrolled 509 patients, the majority of whom had previous therapeutic incursions exposition, either to chronic lymphocytic leukemia and/or to Richter syndrome.

**Table 1:** Richter syndrome treatment approaches in early clinical trials.

Global approach	Regimen	Phase	Population	Previous treatments	Survival outcomes	Adverse events	Reference
Classical chemotherapeutic agents	HyperCVXD	II	29 patients (males=23). Median age at enrollment of 61 years (36–75).	23 patients had fludarabine-based and 2 cladribine-based treatments. 2 out of 11 who achieved CR were TN.	Median survival of 10 months in all patients (19 in those with CR)	Grade 4 granulocytopenia occurred in all cycles. Fever occurred in 24% of the cycles, pneumonia in 15% and sepsis in 8%.	(13)
	FACPGM	II	Total of 22 patients (males=16), of whom 15 with RS. Median age of 62 years (42–74). Median time from CLL diagnosis to RS was of 7.2 years (0.7 – 15.5).	All patients in trial previously treated: 18 had received fludarabine-based treatments, 10 were treated with hyper-CVXD.	Median survival of 2.2 months.	Fever occurred in 41% and infections in 55%. Other AE ≥ G3: anaemia in 62% of cycles, thrombocytopenia in 83% and granulocytopenia in 90%.	(14)
Immunochemotherapy	OFAR I	I-II	Total of 20 patients (males=10). Median age of 66 years (41-78 years).	Only 2 patients had not undergone previous treatments.	6-month survival rates of 59% (53% if del(17p) present).	Anemia, granulocytopenia and thrombocytopenia – with transfusion requirements.	(15)
	OFAR II	I-II	Total of 31 patients (treated with recommended dose).	Median number of previous therapies of 3 (0–9).	Median survival of 6.6 months. 2-years survival rate of 19.7%. Median FFS of 3 months (1.6-4.8). 1-year FFS rate of 17%.	Cytopenia (anaemia, neutropenia, thrombocytopenia), neutropenic fever and infections.	(16)
	R-hyper-CVXD alternating with R-MTX-AraC	II	30 patients with RS enrolled. Median time from CLL diagnosis to RS was of 52 months (0–112).	Pretreated patients.	12-month survival rate of 28%.	Systemic fungal and bacterial infections. Grade 4 neutropenia in all cycles; grade 4 thrombocytopenia in 40% and sepsis ≥ G3 in 20%.	(17)
	R-CHOP	II	15 patients enrolled. Median time from CLL diagnosis to RS of 8.5 years (0 – 17). Unmutated IGHV gene in 8 patients and del(17p) in 4.	11 out of 15 patients previously treated for CLL. Median number of therapeutic incursions of 2 (0–4).	Median PFS of 10 months and median OS of 21 months.	Cytopenia and infections were the most common.	(18)
	CHOP-O	II	37 evaluable patients. Mutated TP53 in 43%.	Median of 1 (0 – 4) prior therapy for CLL (more than 50% with a fludarabine/cyclophosphamide-based regimen).	Median PFS of 6.2 months. Median OS of 11.4 months. Worst outcomes in mutated TP53.	Cytopenia, infections and sepsis.	(19)
	Obinutuzumab, HDMP and lenalidomide	I	6 patients with RS-DLBCL. Median age of 69 years (54–84). 2 cases with complex karyotype and del(17p). Unmutated IGHV in 3 cases.	2 patients without previous treatment (either for CLL or RS).	Patient in CR underwent alloSCT.	Most common AEs ≥ G3 were neutropenia and pulmonary embolism.	(20)
	R-CHOP and blinatumomab (BLINART trial)	II	Median age of 66 years. Del(17p) or TP53 mutations in more than 60% of evaluated patients. 39 patients enrolled, 9 with CR after R-CHOP and not exposed to blinatumomab. 25 patients with blinatumomab induction.	Median of 2 previous therapeutic approaches for CLL (0–7).	Data cut-off of the reports: 1^st^ June of 2022. Without survival outcomes reported.	CRS in 16% of cases. 16% presented with neurotoxicity after blinatumomab exposure, although ≥ G2 in only 2 patients.	(21)
Immunotherapy alone	Pembrolizumab	II	9 patients with a median age of 69 years (46–78). Del(17p) in 3 patients and TP53 mutated in other 2.	All previously treated (median of therapeutic incursions of 5). 5 exposed to purine analogues and 6 to ibrutinib.	Median OS of 10.7 months (95% CI, 4.4-not reached). Median PFS of 5.4 months (95% CI, 2.8-12.2). Increased expression of PD-L1 and PD-1 associated with better outcomes.	Treatment-related AEs≥ G3 were cytopenia (anaemia, neutropenia and thrombocytopenia).	(22)
	Pembrolizumab	II	21 patients with RS-DLBCL subtype (2 with cHL-v excluded from analysis).	Most patients with at least 2 prior treatment regimens for RS.	Median PFS of 1.6 months (95% CI, 1.0–2.1) and OS of 3.8 months (95% CI, 1.8–18.1) – data concerning 25 patients (2 with RS-cHL).	AEs reported in more than 50% of patients, with anaemia, hypothyroidism, fatigue, pyrexia and rash being more common. 6 patients with immune-mediated AEs.	(23)
	Blinatumomab *	II	9 patients enrolled: 8 with complex karyotype, 5 with documented del(17p) and 5 with mutated TP53.	All patients had received prior treatments for CLL (median of 4). 6 out of 9 patients had been previously treated for RS.	Median PFS of 1.9 months and median OS of 10.3 months. 1 patient with CR and another with PR but progressed after 6^th^ cycle.	Most common AE was reversible neuropathy. CRS (GI-II) in 3 patients.	(24)
	Glofitamab	I	10 enrolled patients in dose-escalation and RP2D. 6 evaluable for efficacy.	Not available for this subset of patients. Overall, the cohort was heavily pretreated.	Not available for this subset of patients.	Overall, in the entire cohort, CRS documented in 50% (G3 in 1.2%), being of note an incidence rate of 71.4% in RP2D. Transient ICANS in 5.3% of all patients.	(25)
	Epcoritamab	Ib/II	10 patients enrolled with a median age of 70.5 years (53–80)	Patients with no more than 1 prior line of therapy for RS. 6 patients received epcoritamab as frontline treatment for RS.	Ongoing: data cut-off on 15^th^ July of 2022. Two patients died from PD. ORR of 60%.	CRS in 90% of patients (grade<3) – 6 needed tocilizumab. Anemia, diarrhea, hypophosphatemia, and thrombocytopenia. No ICANS reported.	(26)
iBTK	Acalabrutinib	I-II	25 patients (12 males). Median age 66 years (58–73). Median time from CLL to RS of 4.5 years (2.7-9.6). Del(17p) in 7 patients and TP53 mutations in 9.	Median of one prior therapy for RS (0–2). 14 patients previously treated for RS (4 with ibrutinib, 5 with CHOP). 9 received ibrutinib before RS diagnosis.	Median DoR of 6.2 months (95% CI 0.3–14.8) and median PFS of 3.2 months (95% CI 1.8–4.0). Progression of disease in 17 patients.	Most common AEs≥ G3 were anemia and neutropenia.	(27)
	Zanabrutinib ^#^ (NCT02343120)	II	13 patients (8 males).	Median of 1 therapy before RS (0–5). Median of therapies after diagnose of RS of 1 (0–3).	Median DoR of 25.4 (0+ to 29.7+); median PFS 17.3 (0.6–32.2+) and median OS of 29.3 months (0.6–33.8+).	Infections and cytopenia. Atrial fibrillation/flutter and tumour lysis syndrome (TLS) not observed.	(28)
	Pirtobrutinib (NCT03740529)	II	57 patients with RS (50 evaluable – 6 undergone curative intents by pursuing alloSCT; 1 did not receive the RP2D).	Median number of CLL-therapies of 2 (0–13) and median number of RS therapies of 2 (0–7). Prior treatment with iBTK in 16 patients with RS and 34 with CLL.	Median study FW of 9.7 months with a median OS of 13.1 months (95% CI, 7.1-NE). Median response FW time of 5.5 months (data cut-off of 31^st^ January 2022) with a median DoR of 8.6 months (95% CI, 1.9-NE).	Neutropenia was the most frequent AE≥ G3.	(29)
Other targeted therapies	Venetoclax	I	7 patients with a median age of 73 years (57–77). Only 1 patient with high BCL-2 expression.	Median number of previous therapeutic incursions of 3 (2–5).	No complete responses. Median time to first response of 100 days.	Cytopenia (anemia, neutropenia and thrombocytopenia).	(30)
	Selinexor	I	5 evaluable patients.	Not reported for RS patients.	Not reported. 1 patient in PR progressed beyond 9 months. 1 patient in PR underwent alloSCT.	The most frequent AE≥ G3 (all patients with other NHL) were cytopenia and hyponatremia.	(31)
	Entospletinib	II	8 patients enrolled. Unmutated IGHV in 6 patients.	Prior therapy with ibrutinib in 5 patients and idelalisib (PI3Kδi) in 3.	Not reported.	Fatigue, diarrhea, and anemia.	(32)
Combinatorial approaches	Ibrutinib → ofatumumab	Ib/II	3 patients	Not specified.	Only 1 patient achieved PR: DoR of 4.6 months.	Considering all patients (beyond RS subgroup), diarrhea, infusion-related reaction, peripheral sensory neuropathy and stomatitis were the most common.	(33)
	Selinexor + ibrutinib	I	8 patients enrolled. No cytogenetic or genomic data available.	All patients previously treated: 7 of whom with previous iBTK; 1 patient with previous alloSCT.	Only 1 patient presented CR. No further data available.	Nausea, diarrhea, fatigue, and anorexia	(34)
	Ibrutinib + nivolumab	II	20 patients with RS (8 male). Median age 67.5 years (56-70.5).	Previously treated with median number of therapeutic incursions of 2 (1–3). All were exposed to alkylating agents and 11 to purine analogues.	Median PFS of 5.0 months for a median FW of 8.7 months. Median OS of 10.3 months for a median FW of 8.9 months. During FW, 11 patients had disease progression or died.	Most common AEs≥ G3 were cytopenia (anemia in 7 patients and neutropenia in 8).	(35)
	Zanabrutinib + tislelizumab ^#^ (NCT02795182)	II	7 patients	All patients with relapsed RS. Median of 3 therapeutic incursions for RS (1–5)	Median DoR of 17.2 months (2.9–48.4); median PFS of 2.9 (2.4–51.4+) and median OS of 15.4 (3.5–51.5+). The patient in CR maintained it for 17.5 months.	AEs≥ G3 were neutropenia, thrombocytopenia and TLS (the former in 3 patients).	(28)
	DTRM-12(iBTK), everolimus and pomalidomide	I	11 evaluable patients with RS.	Median of 5 prior therapies. 7 patients with prior iBTK exposure, 3 to PD-1 mAb, 4 to anti-CD20 mAb and 3 to cell-based therapies (CAR-T/stem cell transplant).	Median DoR of 15 months as of the data cut-off (for patients with RS and DLBCL).	Thrombocytopenia, neutropenia and anemia	(36)
	Umbralisib + ublituximab + pembrolizumab	I	4 patients evaluable.	Previously treated patients. Responders were ibrutinib refractory and had up to 8 prior therapeutic incursions (including cell-based therapies).	2 patients with CR maintained at data cut-off: more than 15 and 7 months of FW.	Beyond the RS subgroup, the most common AEs≥ G3 were neutropenia, ALT/AST increase and hypophosphatemia.	(37)
	Venetoclax and daR-EPOCH	II	26 patients with a median age of 63 years (20 in the extension cohort). 52% with complex karyotype and 42% with TP53 mutated.	Median of 1 prior therapy for CLL (0–7) - 41% exposed to ibrutinib, 22 to venetoclax and 15% to PI3Ki. 2 patients previously treated for RS. 6 patients never treated before.	Median FW of 17 months, with a median PFS of 10.1 months and median OS of 19.6 months. Among responders 8 underwent alloSCT and 1 therapy with CAR T cells.	Neutropenia, thrombocytopenia and febrile neutropenia.	(38)
	Venetoclax + obinutuzumab + atezolizumab	II	7 patients newly diagnosed with median age of 70 years (52–80), in whom 6 presented unmutated IGHV; and 3 displayed TP53 mutation and 2 a NOTCH1 mutation. 1 patient with R/R RS.	Only one had not received treatment for CLL. No previous venetoclax exposure.	Median FW of 11.2 months. Among responders, 3 underwent allo-SCT at 4.1, 4.2 and 6.6 months.	Pancreatitis and diabetes mellitus reported in 1 patient.	(39)
Cell-based therapies	Anti-CD19 CAR-T cell (ARI-009)	II	6 patients enrolled (5 evaluated). 3 patients with unmutated and 1 patient with mutated IGHV, respectively (other not assessed). Del(17p) or TP53 mutated in 4.	All patients heavily pretreated. One of them with previous alloSCT.	Patients with CR were disease free with a median PFS of 12.5 months (1.4-26.7), for a median FW of 5.6 months, 2 patients without CR relapsed with CD19 negative RS.	CRS in 4 patients (G<3) and ICANS in none.	(40)
	Anti-CD19 CAR-T cell	I-II	5 patients enrolled with a median age of 65 years (47–70). Del(17p) in 3 patients; 1 patient with complex karyotype.	Pretreated patients (all presented disease progression upon ibrutinib).	Not reported specifically in this subset of patients.	CRS in 2 patients (G<3). ICANS G3 in 1 patient.	(41)
	Anti-CD19 CAR-T cell	II	8 patients enrolled, 6 with RS-DLBCL subtype. Median age of 63.5 years in the former (62, 73). Median time of 8 years from CLL diagnosis to RS (1–16)	Patients with disease transformation after iBTK and/or BCL2 inhibitors. Median of 3 (0–5) therapeutic incursions in CLL and 2 (1–3) in large cell lymphoma.	Median FW of 5.5 months in responders (4, 10). 2 patients underwent alloSCT with curative intent.	5 patients with CRS, of whom a case of G4. 2 ICANS (1 patient with G4).	(42)

CHOPO, cyclophosphamide, doxorubicin, vincristine, prednisone and Obinutuzumab; c-HLv, classic Hodgkin lymphoma variant of Richter Syndrome; CLL, chronic lymphocytic leukemia; CR, complete response; daR-EPOCH, dose-adjusted etoposide, prednisone, vincristine, cyclophosphamide, doxorubicin and rituximab; DoR, duration of response; FACPGM, fludarabine, Ara-C, cyclophosphamide, cisplatin and granulocyte-macrophage colony-stimulating factor; FFS, failure-free survival; HDMP, high-dose methylprednisolone; HyperCVXD, cyclophosphamide, vincristine, liposomal daunorubicin, and dexamethasone; OFAR, oxaliplatin, fludarabine, cytarabine, and rituximab; OS, overall survival; PFS, progression-free survival; R-CHOP, rituximab plus cyclophosphamide, doxorubicin, vincristine, and prednisone; RP2D, recommended phase 2 dose; TN, treatment naïve.

* In the BLINART trial, patients underwent R-CHOP debulking. If complete response was achieved after two cycles, they did not progress to blinatumomab, contrarily to those ones experiencing partial response. Regarding the trial design, R-CHOP responders were analysed separately from the cohort of partial responders to debulking who were treated with R-CHOP and bispecific antibody thereafter.

# Patients in the trial were treated with zanabrutinib ± tislelizumab, herein analysed separately.

### 3.3 Outcomes of efficacy of therapeutic regimens

Considering the studies reporting objective response rates in patients with RS, the pooled estimate rate was 45.05% (95% CI: 36.93–53.28%; I^2^ = 59% - **Figure 2**). The subgroup analysis revealed higher overall response rates upon chemoimmunotherapy, iBTK-based and combination treatment approaches (iBTK ± immune checkpoint inhibitor ± PI3K inhibitor ± immunochemotherapy backbones ± other target agents). In fact, overall response rates were 45.97% (95% CI: 37.75–54.29%; I^2^ = 0%) for chemoimmunotherapy, 51.14% (95% CI: 40.37–61.85%; I^2^ = 0%) for iBTK-based approaches and 53.20% (95% CI: 36.75–69.37%; I^2^ = 41%) for combination therapy. Chemoimmunotherapeutic approaches outlasted classical chemotherapeutic regimens, which presented modest objective response rates of 29.13% (95% CI: 6.81–58.02%; I^2^ = 84%). Other targeted therapies beyond iBTK achieved objective responses in 43.66% of patients (95% CI: 18.02-70.82; I^2^ = 0%), yet these agents were unable to produce complete responses.

**Figure 3:**
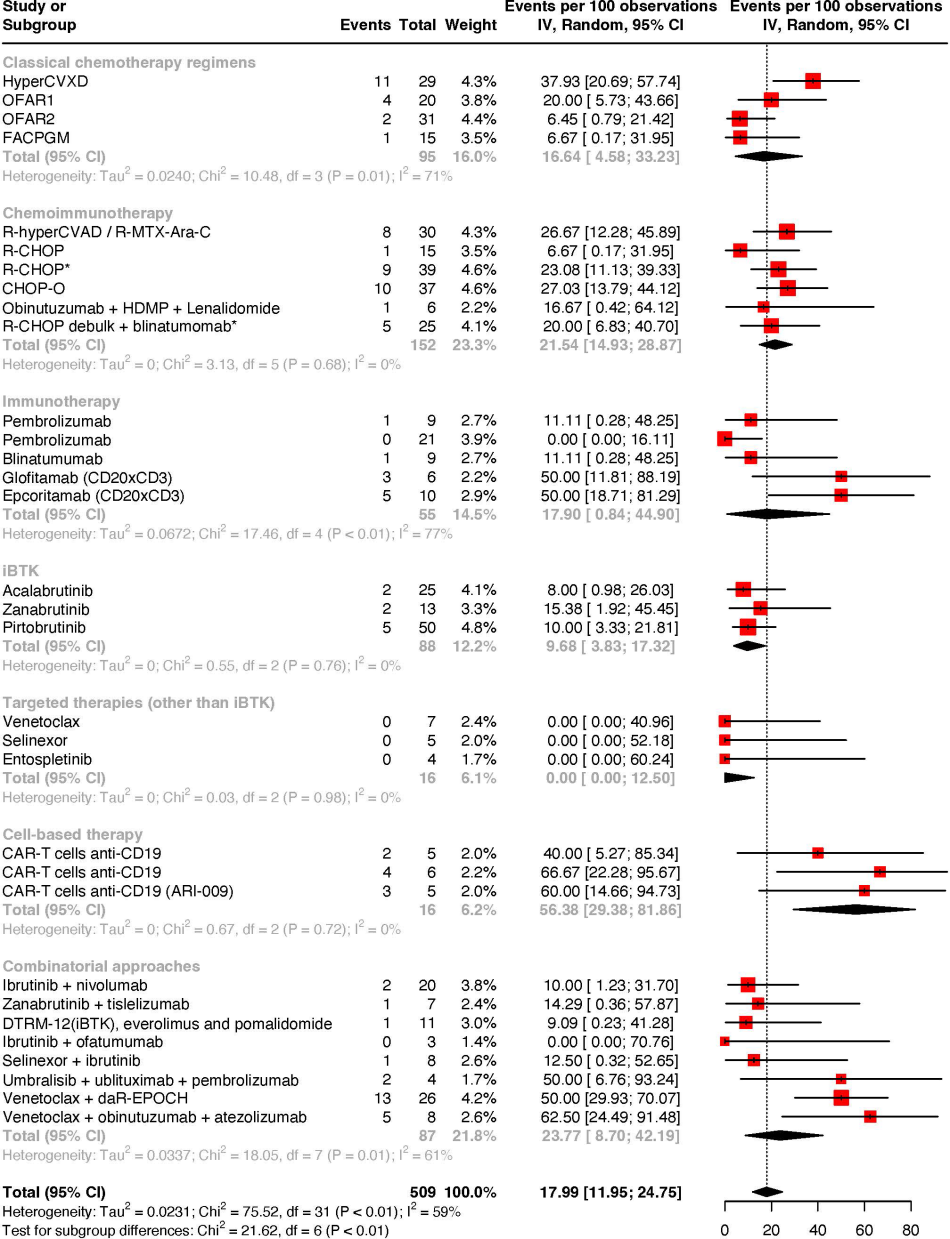
Meta-analysis of complete response rates by treatment strategies.

Regarding complete response rates, pooled estimates were 17.99% (95% CI: 11.95–24.75%; I^2^ = 59% - **Figure 3**). Chemoimmunotherapy regimens achieve higher scores with an overall complete response rate of 21.54% (95% CI: 14.93–28.87%; I^2^ = 0%). Immunotherapy-based strategies achieved a complete response rate of 17.90% (95% CI: 0.84–44.90%; I^2^ = 77%), yet at the expense of a non-negligible heterogeneity.

Cell-based therapies with CD19-directed CAR-T cells achieved the highest pooled scores with a complete response rate of 56.38% (95% CI: 29.38-81.86; I^2^ = 0%), and an overall response rate of 69.01% (95% CI: 41.92 - 91.48; I^2^ = 0%).

Among all the patients with Richter syndrome enrolled in early-phase clinical trials, 54.95% (95% CI: 46.72-63.07) failed to achieve at least a partial response (**Supplementary Figure 3**).

## 4 Discussion

Considering the low prevalence of Richter syndrome, its biological heterogeneity, and its typical stringent clinical urgency demanding a prompt therapeutic approach, the conceptualization and operationalization of randomized clinical trials is a challenge. Despite early trials lacking randomization and the potential for a theoretical selection bias (including patients with milder forms of the disease who can adhere to a feasible treatment timeline, as opposed to heavily pretreated individuals with limited treatment avenues beyond clinical trials), our review acknowledges the valuable insights already present within the analyzed studies, summarizes the evidence and proposes a new conceptual sequential framework of treatment regimens in this cohort of patients.

### 4.1 Classical (immuno)chemotherapy regimens

The sole application of classical chemotherapeutic backbones is outdated, given the burst and continuous expansion of immunotherapeutic and targeted approaches that were able to improve therapeutic outcomes while simultaneously sparing undesirable toxicities. Notwithstanding, the initial trials with classic chemotherapeutic agents were essential for the refinement of modern therapeutic armamentariums. The OFAR 1 and 2 (oxaliplatin, fludarabine, cytarabine, and rituximab) trials explored the role of platinum agents (15); or fludarabine and cytarabine (16), respectively, by means of progressively scaling up the cumulative doses of those agents until undesirable toxicities. Interestingly, OFAR1 achieved higher rates of complete responses that rounded 20% in comparison with 6% in OFAR2. The potential role of platinum agents in RS rendered the substratum to R-DHAP recommendation (rituximab, dexamethasone, cytarabine, cisplatin) as a potential salvage strategy in RS refractory to R-CHOP (1). Besides this proposal regarding R-DHAP as a potential salvage regimen, no specific trials were performed, nor retrospective observational studies are available to reinforce these recommendations. HyperCVXD, although paradoxically associated with the higher rate of complete responses in the classical strategy cohort, was associated with lower rates of objective responses per comparison with other strategies (13). These findings may potentially illustrate the role of a dose-dense cyclophosphamide protocol in a disease with complex biology and heterogeneous behavior. As a matter of fact, we can speculate that although in a subset of patients, this protocol is able to eradicate disease, in other ones, the immune-pauperization induced by cyclophosphamide with a decrease in the counts of immune effector cells may promote the maintenance of disease by sustaining a nurturing microenvironment. Up to date, apart from palliative metronomic chemotherapy protocols, no trials have explored the role of low doses of cyclophosphamide, now acknowledged for paradoxically boosting immune effector functions (43). Herein, cyclophosphamide is a double-edged sword in treating lymphoid malignancies, without a direct relationship between dose and the achieved effect: its use should be titrated in contexts where there is a probable need to manipulate the immune system for cancer treatment.

Concerning R-CHOP, results are different between trials, potentially reflecting different subsets of patients enrolled. Langerbeins et al. demonstrated a reduced response to the abovementioned strategy, although only 15 patients were enrolled, of whom at least 8 displayed adverse prognostic genetic features (18). On the other hand, a subgroup analysis of the BLINART cohort, in whom patients were treated with R-CHOP and only if a partial response was achieved after two cycles, subsequently underwent therapy with blinatumomab (bispecific antibody targeting CD19 in neoplastic cells and CD3^+^ in T cells); initial debulking strategy displayed an objective response rate of 48.7% and a complete response rate rounding 23.0%, considering the 39 patients firstly enrolled (21). Anthracycline-based chemotherapy backbones are particularly interesting given the immunogenic cell death induced in neoplastic tissues, which contrasts with the general state of immunosuppression classically induced by other chemotherapeutic drugs (44).

R-CHOP results as a standalone are not statistically different from those produced by the combination of a CHOP backbone with obinutuzumab, followed by a maintenance strategy with the anti-CD20 antibody after chemotherapy each 8 weeks (19).

### 4.2 Immunotherapy

Considering the use of bispecific antibodies as a standalone strategy or coupled with a sequential debulking strategy first, the last option seems to produce better results. Indeed, considering the R-CHOP debulking followed by blinatumomab immunotherapy, complete response and objective response rounded 20% and 36%, respectively (21); per comparison with blinatumomab alone where more modest response rates of 11% and 22%, respectively (24), were achieved.

In our meta-analysis, besides the reduced number of included patients, when considering the immunotherapeutic strategies as a standalone, patients treated with bispecific antibodies achieved better outcomes per comparison with those treated singly with anti-PD1 (programmed-death 1) antibodies. Bispecific antibodies targeting CD20 in malignant cells produced higher rates of responses (25, 26) per comparison with blinatumomab. Unfortunately, disease relapse after bispecific antibody treatment was biologically characterized not infrequently by the trogocytosis of the targeted differentiation-cluster in the neoplastic entity (24).

### 4.3 iBTK and targeted agents

Bruton-tyrosine kinase inhibitors achieve only modest response rates. Namely, the higher response rates are achieved by zanabrutinib, followed by pirtobrutinib. These results may reflect intrinsic characteristics of the disease, given that the majority of RS enrolled patients was pretreated (for CLL and/or RS), and herein potentially exposed to iBTK and prone to acquire resistances to this targeted therapy or even develop gain of function mutations in downstream signaling mediators. Indeed, patients with CLL who exhibit early refractoriness to iBTK appear to have a higher likelihood of histologic transformation, regardless of acquired resistance to this group of agents. Conversely, those who progress later during therapy often exhibit a higher incidence of variants in the BTK at residue C481 or downstream signaler phospholipase C gamma 2 (PLCγ2) within its autoinhibitory domain, which confers resistance to ibrutinib (45). Besides the BTK variant affecting the activity of iBTK covalent inhibitors (ibrutinib, acalabrutinib and zanabrutinib); noncovalent inhibitors as pirtobrutinib also have variants in BTK kinase domain able to confer resistance (V416L, A428D, M437R, T474I, and L528W), that once again, may be coupled with gain of function variants in PLCγ2 (46). BTK kinase domain variants are documented to appear up to nine months before overt clinical resistance and their search may allow the reshape of the treatment strategy. Moreover, gain-of-function variants of PLCγ2 are dependent on SYK and LYN signaling, with *in vitro* studies showing that the inhibition of the former kinases may counter-regulate the resistance induced by PLCγ2 (47). Metabolic reprogramming with increased oxidative phosphorylation and increased independence upon BTK downstream signaling may further explain iBTK resistance in the setting of RS pathobiology (48).

The administration of iBTK in individuals whose immune function is impaired either by disease progression or by successive cycles of chemotherapy-induced immunosuppression followed by immune-reconstitution phenomena and increased immune-tolerance arousal renders another pathway that might potentially explain the below-average results in this subset of patients. Although previous studies in relapsed or refractory CLL have shown that ibrutinib exposure led to a decrease in pathological B cells, regulatory T cells and myeloid-derived suppressor cells, while preserving naive T cells and NK cells and boosting its activity (49), this parallel immune outcome may become compromised if there is an impairment of the basic physiological immune reserve imposed by overt disease burden and bone marrow involvement.

Overall, the second generation iBTKs, by means of a higher potency and lower off-target effects, coupled with a better pharmacokinetic profile, may explain a mild increase in the efficacy results observed. For instance, zanabrutinib, the drug achieving better results, was shown to display a low affinity to glycoprotein-P, herein displaying lower pharmacokinetic variability (50).

iBTK treatment coupled with anti-PD1 or anti-PD-L1 (programmed-death ligand 1) antibodies seemed to not significantly improve outcomes in treatment response. Once again, heavily pretreated individuals and immune exhaustion may explain the results.

Beyond iBTKs, none of the other targeted agents included in early phase trials (venetoclax – BCL-2 inhibitor; entospletinib – spleen tyrosine kinase inhibitor; and selinexor - selective inhibitor of nuclear export) were able to induce complete responses. The rising trend of treating CLL patients upfront with venetoclax-based regimens may partially account for the results. This is due to the propensity for the development of variants that reduce venetoclax’s affinity towards BCL-2, as well as the upregulation of the anti-apoptotic members BCL-xL and MCL1, with the former being correlated with the overexpression of NOTCH2. In *in vitro* experiments, it was demonstrated that MCL1 inhibitors partially overcame venetoclax resistance when used in combination (47).

### 4.4 Combinatorial approaches to treatment

Among combinatorial approaches, higher response rates are achieved with venetoclax associated with obinutuzumab and atezolizumab, where complete response rounds 62.50% and objective response 87.50%, despite the small number of patients (whose majority was pretreated) enrolled in the early trial (39). Contrarily to CLL cells and non-clonally related DLBCL, PD-1 expression seems to be increased in RS cells. Although more infrequently, PD-L1 may also become expressed in RS and microenvironmental immune players as histocytes and dendritic cells (51). Perhaps, the divergence between the objective response rates of PD-1 monoclonal antibodies (pembrolizumab) as a standalone therapeutic approach and the respective complete response rates act as a reflection of neoplastic heterogeneity within the treated patients, herein imposing a further need in the characterization of the neoplasia before treatment. Assessing the expression levels of PD-1 and PD-L1 in RS may become of surmount importance when defining salvage regimens that may take into consideration the resource to checkpoint inhibitors.

The trial addressing the combinatory regimen with umbralisib (PI3K-δ inhibitor), ublituximab (anti-CD20 mAb) and pembrolizumab enrolled only four patients with RS and produced an ORR and CR of 50%. Umbralisib, beyond PI3K-δ inhibition, downregulates CK-1ε (casein kinase-1 epsilon), allowing a simultaneous sparing the circulatory T regulatory cells (expected to antagonize immune adverse effects mediated by PI3K inhibition) (52).

Lastly, venetoclax associated with daR-EPOCH is another combinatorial regimen producing above-average results in our meta-analysis. Although the majority of patients was previously exposed to venetoclax before enrolment in the trial (38), daEPOCH backbone differs from the CHOP standard by means of a modified drug dosage associated with the addition of etoposide. Beyond topoisomerase II inhibition, etoposide-quinone (metabolite of the drug) is known to impair the activity of CREBBP (acetyltransferase enzyme also with tumour suppressor functions) (53) herein potentially increasing the risk of additional molecular anomalies that increase even further the neoplastic phenotypic aggressiveness. Although the inclusion of etoposide in metronomic palliative regimens is acceptable, the switch of frontline R-CHOP regimens to daEPOCH backbones, may induce more harm than good by increasing the genomic anomalies within tumours with higher cellular turnover, ultimately rendering further chemoresistance.

Zilovertamab-vedotin is an antibody-drug conjugate that by targeting the receptor tyrosine kinase-like orphan receptor 1 (ROR1) in the cellular membrane of neoplastic cells and acting as a cargo for monomethyl auristatin E (antimicrotubule cytotoxic agent), is currently being explored in the context of RS. Although early results have prompted objective responses in four out of seven patients enrolled (54), the inclusion of RS patients both with DLBCL and cHL-variants without a subgroup analysis in preliminary results led to the withdrawal of this agent from our meta-analysis.

## 5 Conclusion and future perspectives – time for a precision medicine approach?

Richter syndrome detrimental prognosis demands a steady hand in disease management and therapeutic sequencing as an attempt to bridge young and fit patients to alloSCT, the only approach up-to-date documented as conveying a curative intent.

After prompt disease immune and molecular characterization, as well as staging, frontline immunochemotherapy with R-CHOP is recommended. In refractory patients or in those who relapse before alloSCT, the therapeutic sequencing should take into consideration the patient and disease characteristics, as well as the previous therapeutic exposures. For instance, fludarabine-based protocols are known to induce immunological dysfunction. For patients with chronic lymphocytic leukemia and treated with RFC (rituximab, fludarabine and cyclophosphamide), a profound depletion of all T-cell populations is documented. Indeed, a median time of 24 months is needed to reach counts of CD4^+^ T levels above 400/μL; while CD8^+^ T cells needed a median time of 12 months to achieve counts within the normal range (55). Analogously, bendamustine is also responsible for inducing a severe T cell depletion, with CD4^+^ T cell counts remaining lower than 200 cells/μL for more than 9 months after treatment completion (56). Given the immune off-target effects produced by these classical chemotherapeutic agents widely used in the treatment of CLL, previous exposition to any of them, especially if recently, should be taken into consideration when defining the therapeutic strategy to RS.

Indeed, in those recently exposed to lymphodepleting chemotherapeutic agents, such as fludarabine or bendamustine, iBTK approaches should be prioritized, particularly in previously naïve patients. Whenever possible, molecular characterization of the neoplasm should be conducted to identify C481 variants of BTK (which confer resistance to covalent inhibitors) or V416L, A428D, M437R, T474I, and L528W (which confer resistance to noncovalent inhibitors). Additionally, a search for pathological variants with gain-of-function mutations in PLCγ2 (which promote disease progression independently of BTK inhibition) should be done. The discovery of any genetic variant impairing the pharmacological inhibition of the Bruton tyrosine kinase pathway should prompt a reconsideration of this strategy. Refractoriness or relapse after iBTK in this setting should trigger the switch of the strategy to an immunotherapeutic approach with bispecific antibodies (bulky disease, higher tumour burden with hyperleukocytosis, or impaired immunological fitness may withdraw this strategy) or a combinatorial approach encompassing venetoclax associated with obinutuzumab and an immune checkpoint inhibitor (dependent on the relative expression of PD1 or PD-L1).

In patients with Richter syndrome arising from a CLL being treated with iBTK, therapy should start with R-CHOP. If the transformation happens early after iBTK incursion, its burden is potentially independent of the BTK resistance-conferring variants, being likely the result of downstream signaler variants with gain of function and subsequent neoplastic cells metabolic reprogramming. In this subset of patients, in case of refractoriness or relapse after R-CHOP, platinum-based protocols (R-DHAP) or a switch to bispecific antibodies can be done and posteriorly, if necessary, an off-label salvage regimen with venetoclax associated with obinutuzumab and an immune checkpoint inhibitor initiated.

In patients with newly diagnosed RS and treatment naïve (either for CLL and RS), following R-CHOP, therapeutic incursions can be performed sequentially with platinum-based protocols (R-DHAP), iBTK, bispecific antibodies and lastly, as an off-label salvage regimen, with a combinatorial strategy encompassing venetoclax associated with obinutuzumab and an immune checkpoint inhibitor.

CAR-T cells produced interesting results, although a small number of patients was assessed by this new cell-based therapeutic approach. Moreover, there is a shortage of extended follow-up data concerning this group of treated patients. However, it is anticipated that as advancements continue in this biotechnological method, modified CAR-T cells might eventually eliminate the need for some patients to undergo alloSCT with curative intent.

## Data availability statement

The raw data supporting the conclusions of this article will be made available by the authors, without undue reservation.

## Author contributions

MS-P: conceptualization, data curation, formal analysis, investigation, methodology, project administration, software, visualization, writing – original draft, writing – review & editing. ÂM: conceptualization, writing – review & editing, data curation. JM: writing – review & editing. PB: conceptualization, project administration, supervision, writing – review & editing.
