# Supplementary material for: Response to therapy in Richter syndrome: a systematic review with meta-analysis of early clinical trials
Source: Front Immunol. 2023 Nov 23;14:1295293. doi: 10.3389/fimmu.2023.1295293 (PMC10702133; doi:10.3389/fimmu.2023.1295293)
Supplement: Supplementary file 1 [file DataSheet_1.docx]

Supplementary Material

# Critical Appraisal Skills Programme (CASP) checklist

**
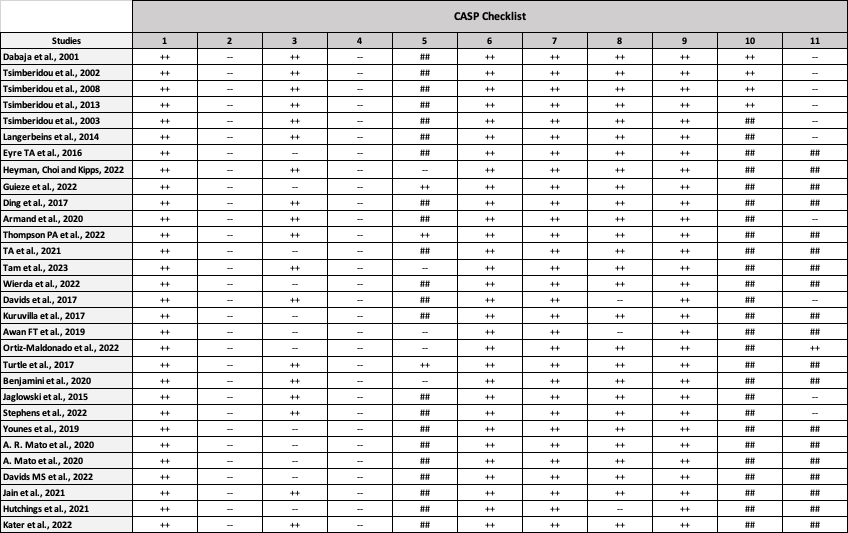
**Table 1: Critical Appraisal Skills Programme (CASP) checklist for clinical trials, adapted due to lack of randomization in the clinical studies included in the meta-analysis.

Questions correspondence as follows:

1. Did the study address a clearly focused research question?
2. Was the assignment of participants to interventions randomised?
3. Were all participants who entered the study accounted for at its conclusion?
4. Were the participants ‘blind’ to intervention they were given? Were the investigators ‘blind’ to the intervention they were giving to participants? Were the people assessing/analysing outcome/s ‘blinded’?
5. Were the participants similar at the start of the trial?
6. Apart from the experimental intervention, did each participant receive the same level of care (that is, were they treated equally)?
7. Were the effects of intervention reported comprehensively?
8. Was the precision of the estimate of the intervention or treatment effect reported?
9. Do the benefits of the experimental intervention outweigh the harms and costs?
10. Can the results be applied to your local population/in your context?
11. Would the experimental intervention provide greater value to the people in your care than any of the existing interventions?

**Codification**: Answers displayed by: ++ (Yes); -- (No); ## (Can’t tell)

# Funnel plots


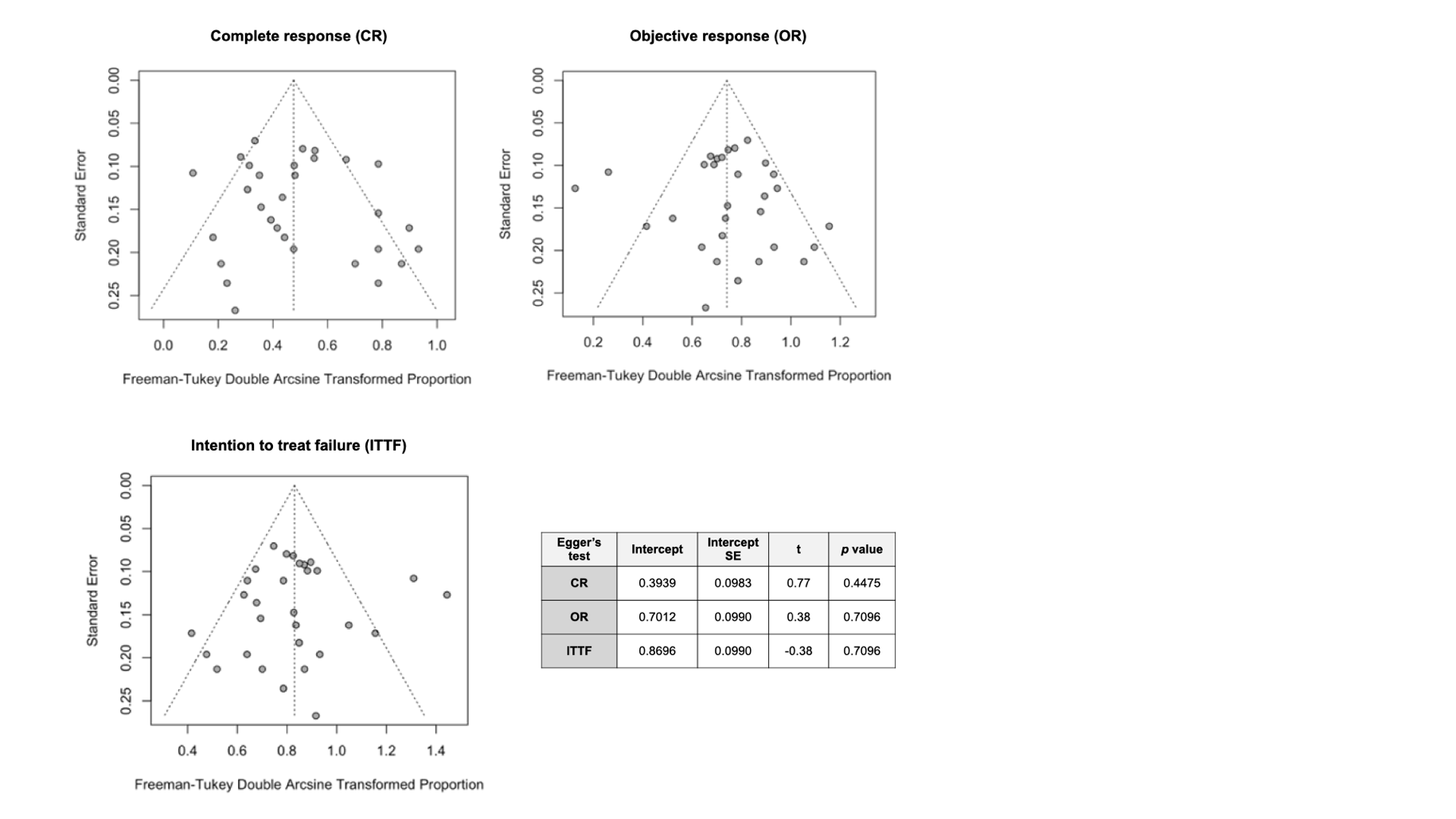


**Figure 2:** Funnel plots illustrate the meta-analysis results for the included studies in the pooled analysis for complete response rate, objective response rate and intention-to-treat failure rate.

Publication bias assessment, according to Egger’s test, suggests the absence of bias (p-value> 0.05 for all outcomes). SE (standard error).

# Intention to treat failure meta-analysis


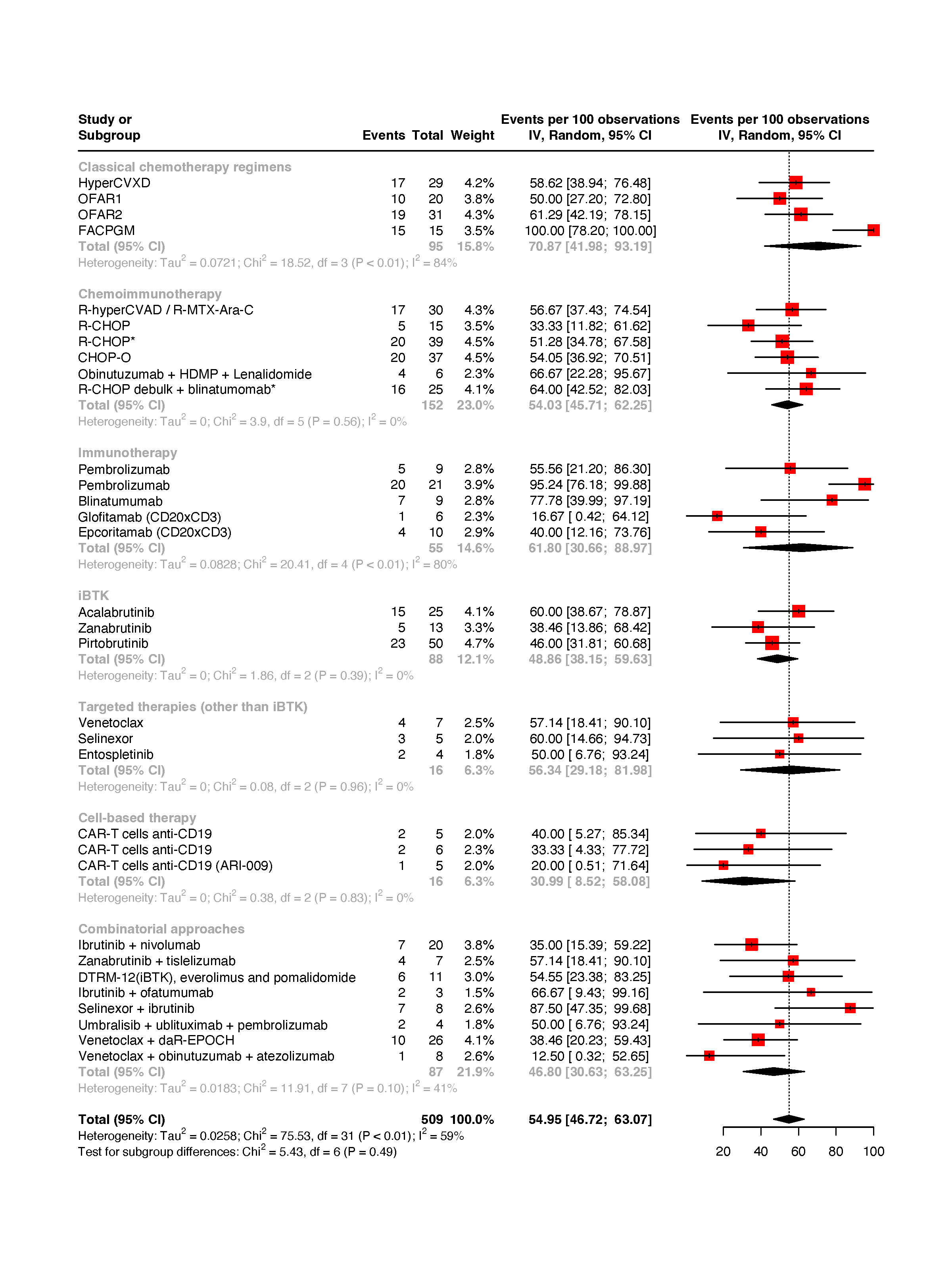


**Figure 3:** Intention to treat failure meta-analysis. Proportions of patients not achieving at least a partial response according to different therapeutic strategies/regimens.
